# Production of poly(GA) in *C9ORF72* patient motor neurons derived from induced pluripotent stem cells

**DOI:** 10.1007/s00401-019-02083-z

**Published:** 2019-10-17

**Authors:** Sandra Almeida, Gopinath Krishnan, Mia Rushe, Yuanzheng Gu, Mark W. Kankel, Fen-Biao Gao

**Affiliations:** 1 Department of Neurology, University of Massachusetts Medical School, Worcester, MA 01605 USA; 2 Neuromuscular and Movement Disorders, Biogen, Cambridge, MA 02142 USA

GGGGCC (G_4_C_2_) repeat expansion in the first intron of *C9ORF72* is the most common genetic cause of amyotrophic lateral sclerosis (ALS) and frontotemporal dementia (FTD) [3, 9]. A key pathological hallmark of *C9ORF72*-related ALS/FTD is the accumulation of dipeptide repeat (DPR) proteins synthesized from both sense and antisense repeat RNAs in affected neurons [2, 8, 14]. The toxicity of poly(GA), poly(GR) and some other DPR proteins in cell and animal models has been well documented, including in mouse models. However, it is poly(GR) whose distribution in patient brains seems to best correlate with neurodegeneration [10, 11].

How DPR proteins are synthesized in human *C9ORF72* neurons is unknown. One widely cited hypothesis is repeat-associated non-AUG (RAN) translation, which posits a novel mechanism by which ribosomes directly initiate translation on expanded repeats [13]. Over time, the term “RAN translation” has evolved to also refer to the phenomenon, that various expanded repeat sequences such as G_4_C_2_ repeats can be translated in different frames. However, whether this truly represents a novel translation mechanism has not yet been vigorously tested in human neurons.

To investigate how DPR proteins are synthesized in *C9ORF72* human neurons, we used CRISPR-Cas9 technology to generate a homozygous deletion in the first intron of *C9ORF72,* 5′ to the G_4_C_2_ repeats (Figs. 1a, S1 and S2) to assess the effect of this deletion on DPR production. This deletion contains a CUG start codon that was shown to play a role in poly(GA) production in reporter constructs [4, 12]. The induced pluripotent stem cell (iPSC) line used in this experiment contains ~ 1000 copies of the G_4_C_2_ repeats [1]. We selected two iPSC lines containing a deletion in intron 1 and differentiated them and the parental iPSC line into ChAT-positive motor neurons (Figs. 1b and S3) as described [5]. This intronic deletion did not compromise the splicing of the intron containing the expanded repeats, as the levels of mature mRNAs for *C9ORF72* variants 1–3 and antisense repeat RNA did not decrease (Fig. 1c–f).

**Fig. 1:**
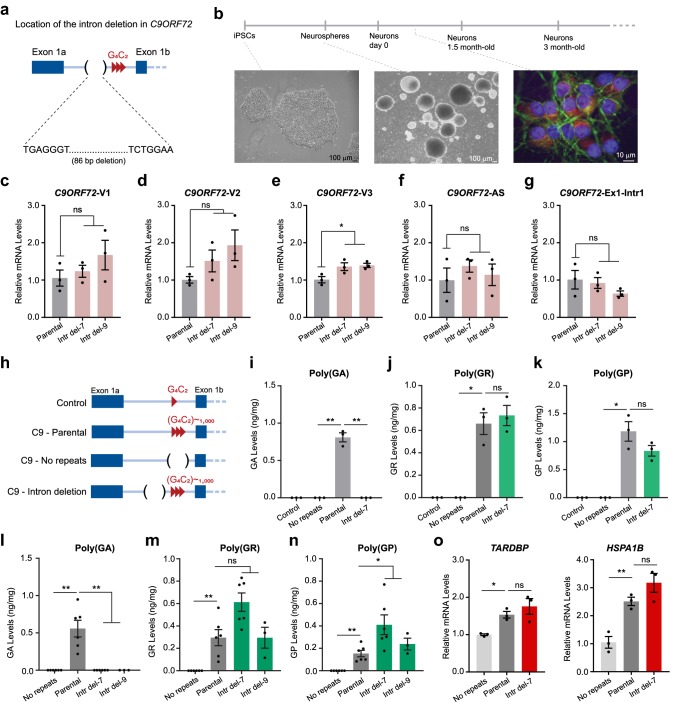
Poly(GA) production in *C9ORF72* iPSC-derived motor neurons. **a** Schematic of the location of the CRISPR-Cas9-mediated deletion in the first intron of *C9ORF72*. **b** Schematic of the motor neurons differentiation protocol. Images show iPSC colonies, neurospheres, and 10-day-old ChAT^+^ (red) and MAP2^+^ (green) neurons (DAPI in blue). **c**–**g** Neurons from parental and intron deletion lines were analyzed for expression of *C9ORF72*-V1, *C9ORF72*-V2, *C9ORF72*-V3, *C9ORF72*-antisense mRNAs, and *C9ORF72* pre-mRNA, *n* = 3 independent differentiations. **h** Schematic of the *C9ORF72* locus in four types of iPSC-derived neurons. **i**–**n** Poly(GA), poly(GR), and poly(GP) levels in 1.5-month-old (*n* = 3 independent differentiations) and 3-month-old (*n* = 3 or 6 independent differentiations) control, parental, and deletion motor neurons measured by MSD immunoassays. **o** Relative expression levels of *TARDBP* and *HSPA1B* in parental and intron deletion lines, *n* = 3 independent differentiations. In all panels, each data point represents one independent differentiation. Values are mean ± SEM. *p < 0.05, **p < 0.01 (**c**–**g** and **o**, one-way ANOVA; **i**–**n**, Welch’s t-test)

To determine whether this deletion affects DPR production, in addition to the intron deletion and the parental iPSC lines described above, an iPSC line derived from a control subject [1] and an isogenic line in which expanded G_4_C_2_ repeats have been deleted by CRISPR-Cas9 [5] (Fig. 1h) were differentiated three times into motor neurons. After 1.5 months, motor neuron cultures were collected and analyzed for poly(GA) content, in a blinded manner, with a new Meso Scale Discovery (MSD) immunoassay developed at Biogen Inc (see Suppl. Information). We found that poly(GA) production was abolished in neurons containing the intronic deletion (Fig. 1i). The absence of poly(GA) production was not due to the absence of the mRNA translation template, as repeat-containing introns were still spliced (Fig. 1c, e) and the level of the pre-mRNA was not significantly decreased (Fig. 1g). More importantly, both poly(GR) and poly(GP) were still produced (Fig. 1j, k). To confirm these results, we differentiated again the no-repeats, the *C9ORF72* parental and the two intronic deletion lines 6 or 3 more times into 3-month-old motor neuron cultures. Once more, blinded poly(GA) measurements confirmed the absence of poly(GA) in intronic deletion lines (Fig. 1l), while poly(GR) and poly(GP) production was not decreased (Fig. 1m, n).

Next, we used this model to investigate the contribution of poly(GA) to molecular phenotypes found in *C9ORF72* iPSC-derived neurons or patient brain tissues. Upregulation of the expression of heat shock proteins and TAR DNA binding protein 43 (TDP-43) have been reported in ALS/FTD patient brain tissues [6, 7]. Here, we show that both *HSPA1B* and *TARDBP* mRNA are upregulated in 4-month-old motor neuron cultures (Fig. 1o). However, in the absence of poly(GA), these molecular phenotypes were not rescued, suggesting that, at least for these phenotypes in this experimental system, poly(GA) was not a key toxic DPR protein.

Our results show that in *C9ORF72* patient neurons, ~ 1000 copies of G_4_C_2_ repeats alone are not sufficient to direct ribosome entry to initiate translation of poly(GA) frame. What remains to be determined is whether other cis elements within the first intron initiate the synthesis of poly(GR) and other DPR proteins or whether their production results from bona fide “RAN translation” in *C9ORF72* patient neurons. These mechanistic investigations will help in the design of therapeutic approaches that aim to decrease DPR protein production.

## Electronic supplementary material

Below is the link to the electronic supplementary material.

Supplementary file1 (DOCX 1052 kb)
