# Supplementary material for: Production of poly(GA) in C9ORF72 patient motor neurons derived from induced pluripotent stem cells
Source: Acta Neuropathol. 2019 Oct 17;138(6):1099–101. doi: 10.1007/s00401-019-02083-z (PMC6851345; doi:10.1007/s00401-019-02083-z)
Supplement: Supplementary file 1 — Supplementary file1 (DOCX 1052 kb) [file 401_2019_2083_MOESM1_ESM.docx]

**Supplementary Information**

**A. Supplementary figures**


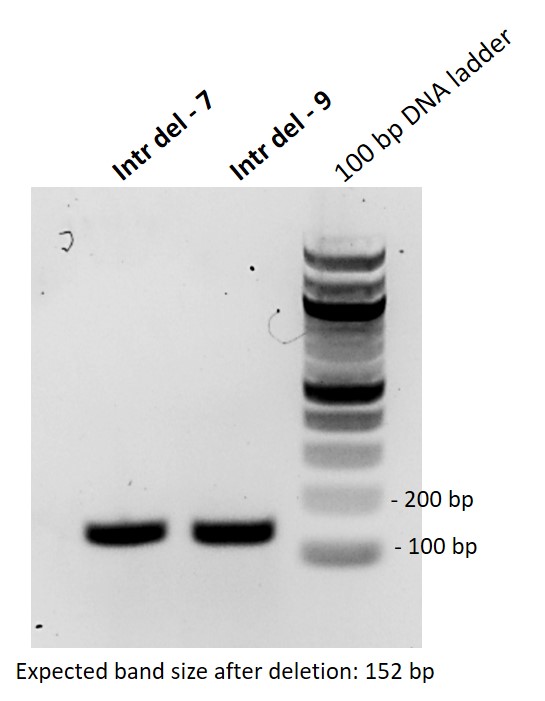


**Supplementary Fig. 1** PCR amplification of the genomic region 5’ to the G_4_C_2_ repeats in the first intron of *C9ORF72*, showing that all lines were homozygous for the deletion.

**Parental (exon 1a/b in grey, repeats in yellow)**

ACGTAACCTACGGTGTCCCGCTAGGAAAGAGAGGTGCGTCAAACAGCGACAAGTTCCGCCCACGTAAAAGATGACGCTTGGTGTGTCAGCCGTCCCTGCTGCCCGGTTGCTTCTCTTTTGGGGGCGGGGTCTAGCAAGAGCAGGTGTGGGTTTAGGAGGTGTGTGTTTTTGTTTTTCCCACCCTCTCTCCCCACTACTTGCTCTCACAGTACTCGCTGAGGGTGAACAAGAAAAGACCTGATAAAGATTAACCAGAAGAAAACAAGGAGGGAAACAACCGCAGCCTGTAGCAAGCTCTGGAACTCAGGAGTCGCGCGCTA(GGGGCC)_~1,000_GGGGCGTGGTCGGGGCGGGCCCGGGGGCGGGCCCGGGGCGGGGCTGCGGTTGCGGTGCCTGCGCCCGCGGCGGCGGAGGCGCAGGCGGTGGCGAGTGG

**Intron deletion – 7: 86 bp deletion**

ACGTAACCTACGGTGTCCCGCTAGGAAAGAGAGGTGCGTCAAACAGCGACAAGTTCCGCCCACGTAAAAGATGACGCTTGGTGTGTCAGCCGTCCCTGCTGCCCGGTTGCTTCTCTTTTGGGGGCGGGGTCTAGCAAGAGCAGGTGTGGGTTTAGGAGGTGTGTGTTTTTGTTTTTCCCACCCTCTCTCCCCACTACTTGCTCTCACAGTACTCGC- - - - - - - - - - - - - - - - - - - - - - - - - - - - - - - - - - - - - - - - - - - - - - - - - - - - - - - - - - - - - - - - - - - - - - - - - - - - - - - - - - - - - - - - - - - - -CTCAGGAGTCGCGCGCTA(GGGGCC)_~1,000_GGGGCGTGGTCGGGGCGGGCCCGGGGGCGGGCCCGGGGCGGGGCTGCGGTTGCGGTGCCTGCGCCCGCGGCGGCGGAGGCGCAGGCGGTGGCGAGTGG

**Intron deletion – 9: 86 bp deletion**

ACGTAACCTACGGTGTCCCGCTAGGAAAGAGAGGTGCGTCAAACAGCGACAAGTTCCGCCCACGTAAAAGATGACGCTTGGTGTGTCAGCCGTCCCTGCTGCCCGGTTGCTTCTCTTTTGGGGGCGGGGTCTAGCAAGAGCAGGTGTGGGTTTAGGAGGTGTGTGTTTTTGTTTTTCCCACCCTCTCTCCCCACTACTTGCTCTCACAGTACTCGC- - - - - - - - - - - - - - - - - - - - - - - - - - - - - - - - - - - - - - - - - - - - - - - - - - - - - - - - - - - - - - - - - - - - - - - - - - - - - - - - - - - - - - - - - - - - -CTCAGGAGTCGCGCGCTA(GGGGCC)_~1,000_GGGGCGTGGTCGGGGCGGGCCCGGGGGCGGGCCCGGGGCGGGGCTGCGGTTGCGGTGCCTGCGCCCGCGGCGGCGGAGGCGCAGGCGGTGGCGAGTGG

**No repeats: ~6Kbp deletion**

ACGTAACCTACGGTGTCCCGCTAGGAAAGAGAGGTGCGTCAAACAGCGACAAGTTCCGCCCACGTAAAAGATGACGCTTGGTGTGTCAGCCGTCCCTGCTGCCCGGTTGCTTCTCTTTTGGGGGCGGGGTCTAGCAAGAGCAGGTGTGGGTTTAGGAGGTGTGTGTTTTTGTTTTTCCCACCCTCTCTCCCCACTACTTGCTCTCACAGTACTCGCTGAGGGTGAACAAGAAAAGACCTGATAAAGATTAACCAGAAGAAAACAAGGAGGGAAACAACCGCAGCCTGTAGCAAGCTCTGGAACTCAGGAGTCGCGC- - - - - - - - - - - - - - - - - - - - - - - - - - - - - - - - - - - - - - - - - - - - - - - - - - - - - - - - - - - - - - - - - - GCGGTTGCGGTGCCTGCGCCCGCGGCGGCGGAGGCGCAGGCGGTGGCGAGTGG

**Supplementary Fig. 2** Sequencing data from each of the *C9ORF72* intron deletion and no-repeats iPSC lines used in this study compared to the parental line.


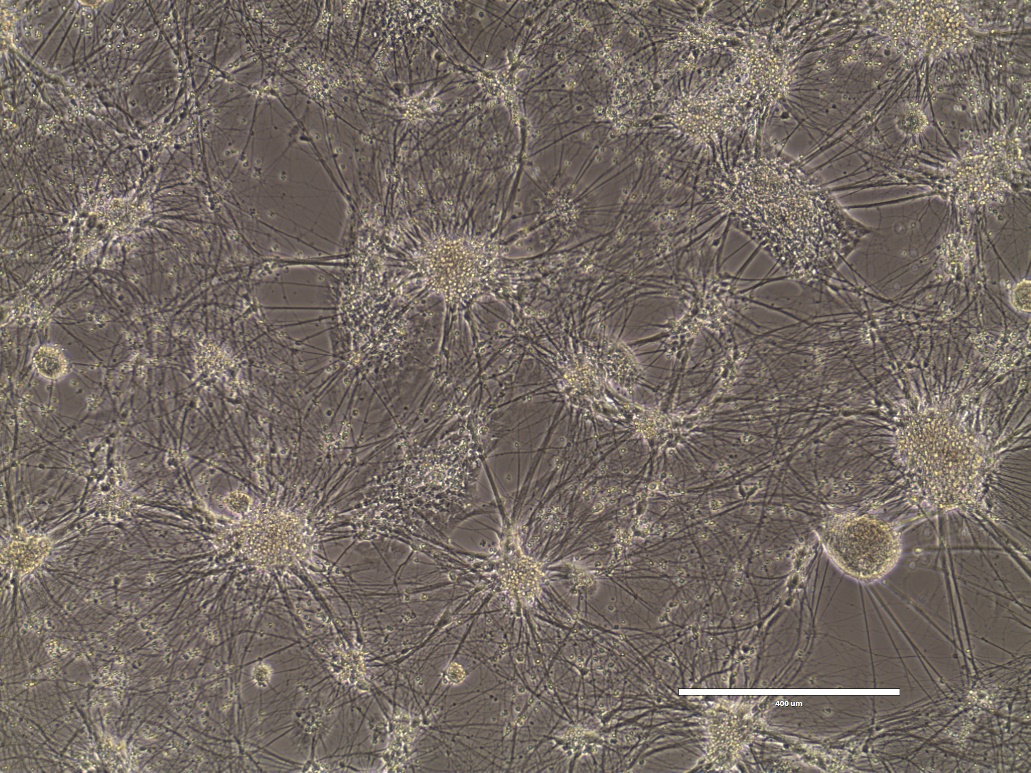


**200 µm**

**Supplementary Fig. 3** Phase contrast image of a 1.5 month-old motor neuron culture.

**B. Supplementary methods**

**Generation of *C9ORF72* intron deletion iPSC lines.** Genome editing with the CRISPR-Cas9 system to create a deletion in the first intron of *C9ORF72,* 5´ to the G_4_C_2_ repeats was performed at ALSTEM (Richmond, CA). Briefly, the Neon electroporation system was used to transfect iPSCs with two different guide RNAs: GCTCTCACAGTACTCGCTGA and TGTAGCAAGCTCTGGAACTC. Single cells were placed in 96-well plates, cultured for 14 days and expanded. Genomic DNA from each clone was extracted with the Zymo genomic extraction kit. A PCR amplification assay was used to identify clones with the desired homozygous deletion, and the PCR products were sent for sequencing. iPSCs were expanded and collected to isolate genomic DNA. The region of interest was amplified by PCR (Supplementary Fig. 1), and the products were sent for sequencing to confirm the identity of each clone (Supplementary Fig. 2). The no-repeats iPSC line was generated and characterized before (Lopez-Gonzalez et al, 2019). The deletion carried by this line eliminated the approximately 1,000 G_4_C_2_ repeats present in the original *C9ORF72* parental line. Sequencing of the flanking regions is provided in Supplementary Fig. 2.

**Motor Neuron cultures.** Motor neurons were differentiated as described before (Lopez-Gonzalez et al., 2016). Briefly, iPSC colonies were seeded on Matrigel-coated wells in mTeSR1 medium (StemCell Technologies); 24 h later, the medium was changed to neuroepithelial progenitor (NEP) medium (1:1 DMEM/F12:Neurobasal, 0.5x N2, 0.5x B27, 0.1 mM ascorbic acid (Sigma), 1x Glutamax, 3 μM CHIR99021 (StemCell Technologies), 2 μM DMH1 (StemCell Technologies), and 2 μM SB431542 (Stemgent)) and replaced every other day for 6 days. Progenitor colonies were dissociated with Accutase, seeded at 1:6 on Matrigel-coated wells, and cultured in NEP medium containing 0.1 μM retinoic acid and 0.5 μM purmorphamine for 6 days; the medium was replaced every other day. Motor neuron progenitors were lifted, cultured in suspension for 6 additional days in the absence of CHIR99021, DMH1 and SB431542 and dissociated to single cells with Accutase. Cells were seeded on poly-lysine/laminin-coated wells in motor neuron medium (1:1 DMEM/F12:Neurobasal, 0.5x N2, 0.5x B27, 0.1 mM ascorbic acid, 1x Glutamax, 10 ng/mL BDNF, 10 ng/mL GDNF, 1 μg/mL laminin, 0.1 μM Compound E, 0.5 μM retinoic acid and 0.1 μM purmorphamine) for up to 4 months. This protocol generated a culture with >90% ChAT^+^ neurons. All experiments were done with neurons derived from 3 (1.5- and 4-month-old cultures) or 6 (3-month-old cultures) independent differentiations.

**Measurement of poly(GR) and poly(GP)**. Poly(GR) and poly(GP) were measured with a Meso Scale Discovery (MSD) immunoassay as described (Choi et al, 2019). Briefly, neurons were lysed in ice-cold RIPA buffer (Thermo Fisher Scientific) containing a cocktail of protease and phosphatase inhibitors (Thermo Fisher Scientific), sonicated on ice at a 20% pulse rate for 15 s and centrifuged at 16,000 *g* for 20 minutes at 4°C. The protein content of supernatants was determined with the Bio-Rad Protein assay reagent (BioRad). Neurons samples (2 µg/µL) were loaded on a 96-well single-spot plate (Meso Scale Discovery; Cat. No. L45XA) pre-coated with a custom-made polyclonal rabbit anti-(GR)_8_ or anti-(GP)_8_ antibodies (1 µg/ml, Covance) and tested in duplicate wells. Serial dilutions of recombinant (GR)_8_ or (GP)_8_ peptide in 1% BSA-TBST were used to prepare the standard curve. The detection antibodies were anti-(GR)_8_ or anti-(GP)_8_ antibodies previously tagged with GOLD SULFO (GOLD SULFO-TAG NHS-Ester Conjugation Pack, Meso Scale Discovery; Cat. No. R31AA) at a concentration of 0.5 µg/ml. Response signals from the assay plate were acquired with a QuickPlex SQ120 instrument (Meso Scale Discovery). For background correction, values from a no-repeats neuron sample was subtracted from the corresponding test samples.

**Measurement of poly(GA)**. Neuron pellets were thawed on ice in approximately 120 µL lysis buffer (1X TBS, pH 7.4, 1 mM EDTA, 1% triton X-100) with protease inhibitor cocktail (cOmplete, Sigma), vortexed and incubated at 4°C for 15 minutes to fully lyse the pellet. Lysed cells were centrifuged at 14,000 RPM for 20 min at 4°C. Total protein concentration of the remaining supernatant was determined using BCA protein assay (Thermo Scientific). Poly(GA) content was measured using a MSD sandwich immunoassay. In this assay, the human/murine chimeric form of anti-GA antibody chGA3 is used as capture antibody, and human anti-GA antibody GA4 along with a SULFO-tag anti-human secondary antibody is used for detection. Poly(GA) concentrations were interpolated from the standard curve using 60X-GA expressed in HEK 293 cells and expressed as ng/mg total protein. For background correction, values from a no-repeats neuron sample was subtracted from the corresponding test samples (all values equal or slightly lower than that were considered zero).

**C. References**

Choi SY, Lopez-Gonzalez R, Krishnan G, Phillips HL, Li AN, Seeley WW et al. (2019) C9ORF72-ALS/FTD-associated poly(GR) binds Atp5a1 and compromises mitochondrial function in vivo. Nat Neurosci 22:851–862

Lopez-Gonzalez R, Yang D, Pribadi M, Kim TS, Krishnan G, Choi SY et al. (2019) Partial inhibition of the overactivated Ku80-dependent DNA repair pathway rescues neurodegeneration in *C9ORF72*-ALS/FTD. Proc Natl Acad Sci U S A. 116:9628–9633

Lopez-Gonzalez R, Lu Y, Gendron TF, Karydas A, Tran H, Yang D, et al. (2016) Poly(GR) in *C9ORF72*-related ALS/FTD compromises mitochondrial function and increases oxidative stress and DNA damage in iPSC-derived motor neurons Neuron 92:383–391
